# Occupational hazards in medium and large scale industrial sectors in Sri Lanka: experience of a developing country

**DOI:** 10.1186/s13104-019-4790-2

**Published:** 2019-11-20

**Authors:** S. M. Arnold, M. S. K. Wickrematilake, R. M. S. D. Fernando, H. M. R. C. Sampath, R. P. P. Karunapema, P. K. B. Mahesh, P. M. Munasinghe, C. J. Denawaka

**Affiliations:** 1 Quarantine Unit, Ministry of Health, Colombo 10, Sri Lanka; 2 Office of Regional Director of Health Services, Matale, Sri Lanka; 3 Office of Regional Director of Health Services, Puttalam, Sri Lanka; 4 Health Promotion Bureau, Ministry of Health, Colombo 10, Sri Lanka; 5 Ministry of Health, Colombo 10, Sri Lanka; 6 Office of Regional Director of Heath Services, Colombo, Sri Lanka; 7 Office of Medical Officer of Health, Battaramulla, Sri Lanka

**Keywords:** Occupational hazards, Occupational health, Hazard control, Industry

## Abstract

**Objective:**

Occupational hazards is an area where many countries have begun to pay more emphasis as it affects the health of many particularly in developing countries. However, documented literature is scarce in this regard although occupational hazards are common in workplaces. The study was carried out with the objective of describing the physical hazards and control measures adopted in the formal medium and large-scale industrial sector in Sri Lanka.

**Results:**

Of the 69 units of the 25 factories, physical hazards detected in the workplaces were; excessive noise (78.3%), poor light (58%), increased temperature (65.2%), and poor ventilation (68.1%). Over 50% of large machinery and 33% of medium-scale machinery were not adequately guarded. Nearly 41% of the machinery were difficult to operate, of them 36.2% had controls in positions which were hard to reach. Of safety measures adopted, only 34.8% had proper demarcation of areas with 28.9% displaying safety signs. Housekeeping was poor in 59.4% and less than 40% had safe storage of raw materials and end products.

## Introduction

Occupational accidents and work-related diseases remain a relatively uncovered domain in global literature. As a result of occupational accidents or work-related diseases globally attribute to more than 2.78 million deaths and 374 million non-fatal injuries [1]. The consequent losses in production and work time amount to nearly 4% of the gross national product even before the twenty-first century without any reduction up to date [2, 3]. Adverse effects of any occupational hazards in medium and large-scale industries would have an effect on many workers who are employed in those [3–9].

In developing countries new industrial zones consisting of medium and large-scale industries are being set up at a rapid pace [10]. Export Processing Zones (EPZs) are defined as “industrial zones with special incentives set-up to attract foreign investors, in which imported materials undergo some degree of processing before being exported again” [11]. A large number of workers especially youths are employed in these industries. Recognition of health hazards is the first step in hazard control. Inspection of workplace is the best source of direct information about potential health hazards [12–14].

While remaining a significant burden throughout the world, the incidence of different types of workplace diseases and injuries varies among regions [5, 15]. Hence region-specific scientific literature could depict the actual burden and hence more useful in planning purposes. The generated evidence on occupational health hazards is not commonly found in literature related to lower middle income settings [16]. Sri Lanka is no exception for this and even what is available mainly focuses on small-scale industries [17]. The aim of the present study was to describe the physical hazards and control measures adopted in the formal medium and large-scale industrial sector in the Export Processing Industrial Zone (EPIZ). The study was conducted in the Biyagama, EPIZ which is one of the main industrial zones in Sri Lanka with medium and large scale factories and would represent these sectors in Sri Lanka.

## Main text

### Methods

A cross-sectional study was conducted in factories at Biyagama Export Processing Zone (EPZ) which is a main Export Processing Industrial Zone in Sri Lanka. Biyagama EPZ has over 20,000 workers in 49 enterprises. Export Processing Zones consists of medium and large-scale industries. Medium-scale industries consist of industries which employ 50–200 employees and large-scale industries consists of more than 200 employees [18]. An Export Processing Zone was selected since the organization of industries in such zones depict similarities in common.

It was decided to include 50% of factories as the sample which amounts to 25 factories. Twenty-five factories were selected representing all major industrial categories using proportionate sampling technique. The factories selected from each category was proportionate to the total number of factories in each category. A checklist was developed using the Delphi technique involving experts in the field who included occupational physician, industrial engineer, public health specialist, safety officer and a factory manager. Two rounds of questionnaires were sent to the above experts and the aggregated responses were shared after each round. Non-participatory observational method was adopted in data collection using the checklist. When administering the checklist, in instances where there was more than one section in a single factory (e.g. manufacturing, sewing, cutting, packaging etc.). (Additional file 1—checklist) It was administered independently in all major sections.

Common physical factors such as light, temperature, noise, space, ventilation were assessed. The measurements were done using calibrated light meter, thermometer and sound level meter. Space and ventilation were assessed using the parameters stipulated by the Sri Lankan Local Government Authority for space and ventilation for factories. It is stipulated that the space allowed for each worker should be not less than 400 cuft and 1/7 of the wall area should contain windows for ventilation. If the factory was air-conditioned the ventilation was taken as satisfactory. Even if exhaust fans were present if the parameters of the Local Government Authority were not met it was considered as unsatisfactory. Sound level above 85 dB over 8 h working period was considered unsatisfactory. Different high noise levels the workers are exposed throughout a normal working day was measured. The Time Weighted Average was calculated using these noise levels and the time that the workers are exposed to them. Adequacy of light level was determined based on the task performed. Period of work-rest was considered in determine the satisfactory temperature level. Measures taken to prevent occupational accidents such as guarding of machinery, housekeeping, proper storage, warning signs, demarcation of areas, emergency evacuation procedures were observed. In addition, state of the immediate working environment and the machinery operating practices was also assessed through the observational checklist. The data collection was done by five medical officers who have experience in workplace inspection. They had received a specific component of training on this domain within their routine training after being assigned to the current posts. There was no significant inter observer variation when assessed for in the pre-testing period. The Observation did not change the behavior of workers since the machine operation was not alterable by workers practices but workers had to adopt to the machinery for operation practice.

### Results

In total, data was collected from 69 units of the 25 selected factories. When there were more than one section in a single factory (e.g. manufacturing, sewing, cutting, packaging etc.) all section were included. This resulted in 69 units in 25 factories. Table 1 summarized the characteristics of the physical environment of the settings.

**Table 1:** Physical environment of the workstation (N = 69)

Factor	Satisfactory		Unsatisfactory		Total	
	No	%	No	%	No	%
Light	29	42.0	40	58.0	69	100.0
Space	25	36.2	44	63.8	69	100.0
Ventilation	22	31.9	47	68.1	69	100.0
Temperature	24	34.8	45	65.2	69	100.0
Noise	15	21.7	54	78.3	69	100.0

N: work units; %: percentage of number of stations

In the physical environment of the workstation, poor lighting conditions was present in 58% of the working units. With regard to noise, the levels were unsatisfactory in 78.3% of the work units (Table 1).

Safety measures, machinery layout and effects of machinery operation were observed. The safety measures of medium and large-scale machinery revealed that 66% of the medium scale machinery had satisfactory machinery guarding. However, in large-scale machinery only 47.8% had adequate guarding measures.

Table 2 summarizes some selected characteristics related to machine operation.

**Table 2:** Machinery operation (N = 69)

Description	Yes		No	
	No	%	No	%
Machinery difficult to operate	41	59.4	28	40.6
Controls hard to reach	44	63.8	25	36.2
Working surfaces are in proper heights	22	31.9	47	68.1
Equipment cause excessive vibration	22	31.9	47	68.1
Mobile equipment are safe	21	30.4	48	69.6
Body exposed to continues or repeated motions of the equipment	18	26.1	51	73.9
Hand tools difficult to operate	15	21.7	54	78.3

N: work units; %: percentage of number of stations

It was revealed that nearly 60% of machinery were difficult to operate and worker had to put additional effort to operate. In more than 60%, the controls were positioned where the operator found it hard to reach. Approximately only 30% units had favourable work-surface heights and safe mobile equipment. In more than one-fifth of the settings, operation of hand tools was difficult (i.e. heavy, hard to squeeze, slippery etc.)

The reasons for unsafe machinery revealed that 30.6% of the machinery were unguarded and in 59.7% the guarding were unsatisfactory. Measures taken to maintain a safe premises are depicted in Table 3. It was found that majority of the working units were categorized as “unsatisfactory” for the six explored domains.

**Table 3:** Measures taken to maintain a safe premises (N = 69)

Factor	Satisfactory		Unsatisfactory	
	No.	%	No.	%
Demarcation of areas	24	34.8	45	65.2
Safety signs	20	28.9	49	71.1
Safe storage of raw materials	25	36.2	44	63.8
Safe storage of end products	23	33.2	46	66.8
House keeping	28	40.6	41	59.4
Emergency evacuation procedures with demarcated safe areas	22	31.9	47	68.1

N: work units; %: percentage of number of stations

### Discussion

The present study revealed the presence of many physical hazards, unfavourable machinery operations and safety measures in many of the work settings. Hence this addresses the dearth of scientific evidence on medium and large scale industries like EPZs and the findings are invaluable for the policy planners. Poor working conditions which are associated with occupational hazards lead to occupational diseases and injuries [8, 19]. In the present study, major physical hazards such as poor or excessive light (58%) and excessive noise (78.3%) were observed in majority of the settings. Noise is one of the more widely and frequently experienced problems of the industrial work environment. Noise induced hearing loss usually progress unnoticed until it beings to interfere with communication posing a serious safety hazard [9, 20]. In addition to these hearing related complications, noise-exposure in occupational settings have bene found to be associated with negative consequences on other systems of the body [4, 6]. It is further associated with the occurrence of higher frequency of other occupational injuries as well [21].

In the present study, excessive temperature (65.2%), poor ventilation (68.1%) and overcrowding (63.8%) were commonly observed in many work settings. Similar findings have bene reported from Philippine [7]. As Sri Lanka is a tropical country the temperature and humidity are naturally high. If there is overcrowding coupled with poor ventilation it would augment the temperature and humidity to a level where the worker may not be able to concentrate on the tasks performed.

Proper guarding of machinery is essential to prevent accidents in an industry. The present study showed that over 33% of medium size machinery had inadequate guarding and this was 52.2% in large machinery. This implies that the workforce is at a constant risk of getting injured by unguarded machinery. It is always important to assess the situation at the workstation where machines are operated. It was found that over 40% of machines were hard to operate and required excessive force which would mainly give rise to musculoskeletal problems. An interesting finding was that in 36.2% the machinery controls were hard to reach. This is a highly unsatisfactory situation since it would not be possible to switch off the machinery in an emergency and would lead to major trauma and/or death of the operator.

In the present study, the application of measures taken to maintain safe premises were found to be unsatisfactory. It was found that only a minority had taken satisfactory measures. Safety measures such as demarcation of areas (34.8%), safety signs (28.9%), safe storage of raw materials (36.2%), end products (33.2%) shows that these important safety measures have not been duly addressed in a majority of industries.

In over 59% of premises the housekeeping was poor. Poor housekeeping too contributes to accidents and also reduce productivity. Emergency evacuation procedures are important especially in large scale industries where large numbers of workers are present in a given shift. The present study found that only in 31.9% of the workplaces there were proper emergency evacuation procedures with demarcated safe areas. This is a highly unsatisfactory condition since in an emergency such as a fire a large number of workers are being exposed to a major hazard.

## Limitations

There were several limitations of the study. The study being conducted in one main EPZ and small sample size may affect the generalisability of the finding to the entire country. Additionally, the medium and large scale industries were taken as one unit and the stratified analysis was not done. This was due to the similarities present in the study settings except for the number of workers. This was given emphasis in interpretation and generalization of the findings. The measures taken to prevent accidents such as guarding were based on observation on non-availability or unsatisfactory level. The findings were conveyed with descriptive statistics and not using any significance testing. Hence the analysis of confounding effects was not done. As this study was an eye-opener on this field, authors believe that even these descriptive findings would be beneficial for policy formulation.

## Supplementary information

**Additional file 1.** Occupational hazards in medium and large scale industrial sectors in the Biyagama Export Processing Zone—Checklist. An observational checklist to obtain data on key physical hazards in the medium and large scale industrial sectors in the Biyagama Export Processing Zone.

## Data Availability

The datasets used and/or analysed are available from the corresponding author on reasonable request.

## Abbreviation

EPZ: Export Processing Zone
