# Supplementary material for: Occupational hazards in medium and large scale industrial sectors in Sri Lanka: experience of a developing country
Source: BMC Res Notes. 2019 Nov 20;12:755. doi: 10.1186/s13104-019-4790-2 (PMC6865055; doi:10.1186/s13104-019-4790-2)
Supplement: Supplementary file 1 — Additional file 1. Occupational hazards in medium and large scale industrial sectors in the Biyagama Export Processing Zone—Checklist. An observational checklist to obtain data on key physical hazards in the medium and large scale industrial sectors in the Biyagama Export Processing Zone. [file 13104_2019_4790_MOESM1_ESM.pdf]

Serial No: ☐

Occupational hazards in medium and large scale industrial sectors
<u>in the Biyagama Export Processing Zone - Checklist</u>

1. Code Number assigned for the Establishment: ……………………………………..

2. Type of industry: ………………………………………………………………….

4. Section …………………………………………

5. Main activity in the section:………………………………

6. Size of industry:
   1. Medium scale ☐ 4. Large scale ☐

7. No of employees: 1. Males ………. 2.Females: ……… 3. Total: ………

8. No of employees per shift: 1. Males ………. 2.Females: ……… 3. Total: ………

9. Premises

   a. Noise Level: ………dB

   b. Light: …………Lux

   c. Ventilation
      Wall area in square feet: …………ft^2

      Open areas (Doors & Windows) in square feet: ………… ft^2

      Ratio of open area: walls ……..

   d. Temperature: ……..^0C

   e. Space

      Length (ft): ……… Width: ……. (ft) Height: ………(ft)

      Volume: …… $cuft^3$

      Number of persons occupying in a routine shift: ……..

10. Housekeeping

		Yes	No	Remarks
1	Is areas well demarcated			
2	Is safety signs adequately displayed			
3	Are raw materials safely stored			
4	Are end products safely stored			
5	Is the premises well maintained			
6	Is emergency evacuation procedures in place with demarcated safe areas			

11. General Housekeeping

1..Satisfactory ☐ 2. Unsatisfactory ☐

Remarks
………………………………………………………………………………………………
………………………………………………………………………………………………
………………………………………………………………………………………………
………………………………………………………………………………………………
………………………………………………………………………………………………
………………………………………………………………………………………………
………………………………………………………………………………………………
………………………………………………………………………………………………

Machinery & safety

12. Use of electricity power: Yes ☐ No ☐

13. Type of machinery Small ☐ Medium ☐ Large ☐

14. Are machinery adequately guarded? Yes ☐ No ☐

Remarks
………………………………………………………………………………………………
………………………………………………………………………………………………
………………………………………………………………………………………………
………………………………………………………………………………………………
………………………………………………………………………………………………
………………………………………………………………………………………………

15. If “No” why is it prone to accidents

		Yes	No
1.	Not covered		
2.	Access not restricted		
3.	Areas not demarcated		
4.	Others (Pl specify)		

Remarks
…………………………………………………………………………………………………
…………………………………………………………………………………………………
…………………………………………………………………………………………………
…………………………………………………………………………………………………

16. Machinery operation

		Yes	No	Remarks
1.	Is machinery difficult to operate			
2.	Are controls hard to reach			
3.	Are working surfaces in proper heights			
4.	Does the equipment has excessive vibration			
5.	Does the equipment cause excessive noise			
6.	Is mobile equipment safe			
7.	Are any parts of the body exposed to continues or repeated motions of the equipment			
8.	Are use of hand tools difficult(hard to squeeze, slippery, heavy etc)			

Remarks
…………………………………………………………………………………………………
…………………………………………………………………………………………………
…………………………………………………………………………………………………
…………………………………………………………………………………………………
…………………………………………………………………………………………………
…………………………………………………………………………………………………
…………………………………………………………………………………………………
…………………………………………………………………………………………………
…………………………………………………………………………………………………
…………………………………………………………………………………………………
…………………………………………………………………………………………………
…………………………………………………………………………………………………
…………………………………………………………………………………………………
…………………………………………………………………………………………………
…………………………………………………………………………………………………
…………………………………………………………………………………………………

Worker safety

17. Work environment

		Yes	No	Remarks
1	Workstation comfortable to perform task (does not require unusual positions/stretching/straining)			
2	Are working area safe (eg. not slippery, adequate light)			
3	Are there protruding objects prone for accidents (handles/knobs/materials etc)			
4	Is areas well demarcated			

Remarks
……………………………………………………………………………………………………
……………………………………………………………………………………………………
……………………………………………………………………………………………………
……………………………………………………………………………………………………
……………………………………………………………………………………………………
……………………………………………………………………………………………………
……………………………………………………………………………………………………
……………………………………………………………………………………………………
……………………………………………………………………………………………………
……………………………………………………………………………………………………
……………………………………………………………………………………………………
……………………………………………………………………………………………………
……………………………………………………………………………………………………
……………………………………………………………………………………………………
……………………………………………………………………………………………………
……………………………………………………………………………………………………

Hazards

Type of hazard	Point of exposure
**Physical**	
**Chemical**	
**Biological**	
**Ergonomical**	
**Other**	
